# The morphology of the mandibular coronoid process does not indicate that *Canis lupus chanco* is the progenitor to dogs

**DOI:** 10.1007/s00435-015-0298-z

**Published:** 2016-01-21

**Authors:** Luc Janssens, Rebecca Miller, Stefan Van Dongen

**Affiliations:** Department of Archaeology, Leiden University, Einsteinweg 2, 2333 CC Leiden, The Netherlands; Service of Prehistory, University of Liège, quai Roosevelt, 1, 4000 Liège, Belgium; Department of Evolutionary Ecology, University of Antwerp, Groenenborgerlaan 171, 2020 Antwerp, Belgium

**Keywords:** Dog, Wolf, Domestication, Morphology, *Canis lupus chanco*, Mandible, Coronoid process

## Abstract

The domestication of wolves is currently under debate. Where, when and from which wolf sub-species dogs originated are being investigated both by osteoarchaeologists and geneticists. While DNA research is rapidly becoming more active and popular, morphological methods have been the gold standard in the past. But even today morphological details are routinely employed to discern archaeological wolves from dogs. One such morphological similarity between *Canis lupus chanco* and dogs was published in 1977 by Olsen and Olsen. This concerns the “turned back” anatomy of the dorsal part of the vertical ramus of the mandible that was claimed to be specific to domestic dogs and Chinese wolves *C*. *lupus chanco*, and “absent from other canids”. Based on this characteristic, *C*. *lupus chanco* was said to be the progenitor of Asian and American dogs, and this specific morphology has been continuously used as an argument to assign archaeological specimens, including non-Asian and non-American, to the dog clade. We challenged this statement by examining 384 dog skulls of 72 breeds and 60 skulls of four wolf sub-species. Only 20 % of dog mandibles and 80 % of *C*. *lupus chanco* showed the specific anatomy. In addition, 12 % of *Canis lupus pallipes* mandibles showed the “turned back” morphology. It can be concluded that the shape of the coronoid process of the mandible cannot be used as a morphological trait to determine whether a specimen belongs to a dog or as an argument in favour of *chanco* as the progenitor to dogs.

## Introduction

The domestication of wolves into dogs is an active topic of research (Boudadi-Maligne and Escarguel 2014; Germonpré et al. 2009; Larson et al. 2012; Morey and Jaeger 2015; Thalmann et al. 2013). Where, when and from which progenitor wolf sub-species dogs originated has been investigated both by osteoarchaeologists (Aaris-Sørensen 1977; Benecke 1987, 1994; Boudadi-Maligne and Escarguel 2014; Huxley 1880; Iljin 1941; Nehring 1888; Rütimeyer 1861; Stockhaus 1965; Studer 1901; Sumiński 1975) and geneticists (Anderson et al. 2009; Ardalan et al. 2011; Axelsson et al. 2013; Brown et al. 2011; Freedman et al. 2014; Gundry et al. 2007; Ho et al. 2005; Irion et al. 2003; Karlsson et al. 2007; Khosravi et al. 2013; Kirkness et al. 2003; Klütsch and de Caprona 2010; Larson and Burger 2013; Leonard et al. 2002; Lindblad-Toh et al. 2005; Ostrander and Wayne 2005; Pang et al. 2009; Savolainen et al. 2002, 2004; Schmutz and Berryere 2007; Schoenebeck and Ostrander 2013; Thalmann et al. 2013; Tsuda et al. 1997; Vaysse et al. 2011; Verginelli et al. 2005; Vila et al. 1999, 2005; Vilà et al. 1993, 1997; Vonholdt et al. 2010; Wayne 2012; Wayne and Ostrander 1999, 2007).

Briefly there are two current views. One group of researchers proposes an origin of dogs after the Last Glacial Maximum (LGM) in Europe and during the Magdalenian, about 18,000 years ago (Thalmann et al. 2013). This evidence is based on genetic research (Ho et al. 2005; Thalmann et al. 2013), and the morphology of canine archaeological remains that is distinctively smaller than those of wolves (Altuna et al. 1984; Boudadi-Maligne and Escarguel 2014; Boudadi-Maligne et al. 2012; Célérier 1994; Célérier et al. 1999; Chaix 2000; Larson and Burger 2013; Leesch et al. 2012; Morel and Müller 1997; Napierala and Uerpmann 2012; Pionnier-Capitan 2010; Pionnier-Capitan et al. 2011; Street 2002).

The other group claims that dogs originated before the LGM, as early as in the Aurignacian and Gravettian and thus 35,000 years ago (Bocherens et al. 2014; Germonpré et al. 2009, 2012; Ovodov et al. 2011; Sablin and Khlopachev 2002). Although genetic analysis has not found any relationship between these old archaeological canine specimens (Thalmann et al. 2013) purported to be domesticated wolves and modern dogs, these researchers suggest that these animals were, however, domesticated, but did not produce surviving offspring (aborted domestication waves) (Germonpré et al. 2012; Skoglund et al. 2011). The arguments to place these pre-LGM specimens in the dog clade are based on morphology alone and mainly on wider and shorter snouts. Drake et al. (2015) have, however, demonstrated that this criterion (shorter and wider snouts) is not useful in distinguishing dogs from wolves and also identified some of the so-called pre-LGM dog fossils as wolves.

Many morphological differences have been described between wolves and dogs in the literature since the eighteenth century (Clutton-Brock 1962; Degerbøl 1961; Nehring 1888; Stockhaus 1965; Studer 1901; Wolfgram 1894). Three morphological methods were used to examine morphological differences:

- The “obvious” visual difference in appearance (morphology, sensu stricto) (Olsen and Olsen 1977).
- The difference in size (morphometry) (Benecke 1987, 1994; Boudadi-Maligne and Escarguel 2014; Napierala and Uerpmann 2012).
- The difference in appearance (form) that cannot be recognized visually with certainty (geometric morphometrics) (e.g., Drake and Klingenberg 2010; Milenkovic et al. 2010; Pionnier-Capitan 2010; Schmitt and Wallace 2012).

The most frequently reported morphological and morphometric differences used to distinguish dogs from wolves are smaller stature and thus smaller anatomical parts (e.g., skull, teeth such as carnassials, etc.), shorter and wider snouts, tooth crowding, larger orbital angles and a “turned back” morphology of the dorsal side (apex) of the vertical ramus of the mandible (coronoid process) (Olsen and Olsen 1977, Fig. 1 and 2, p. 534–535). The latter morphological difference is based purely on difference in shape. This distinctive morphological characteristic was described in 1977 by Olsen and Olsen (Olsen and Olsen 1977). The authors state that the origin of Asian and American (New World) dogs must have originated in the Far East and proposed the Tibetan wolf (Chinese wolf, Asian wolf, *Canis lupus chanco*) as the dog’s ancestor (Olsen and Olsen 1977, 534). This opinion was based on the specific “turned back” morphology of the coronoid process of the mandible, claimed to be “specific to domestic dogs” and Chinese wolves, and to be “absent from other canids” (Olsen and Olsen 1977, 534). Based on this assumption, *C. lupuschanco* was said to be “the progenitor of dogs”, and this specific morphological trait is still used in recent publications to assign archaeological specimens to the dog clade (e.g., Ovodov et al. 2011).

**Fig. 1:**
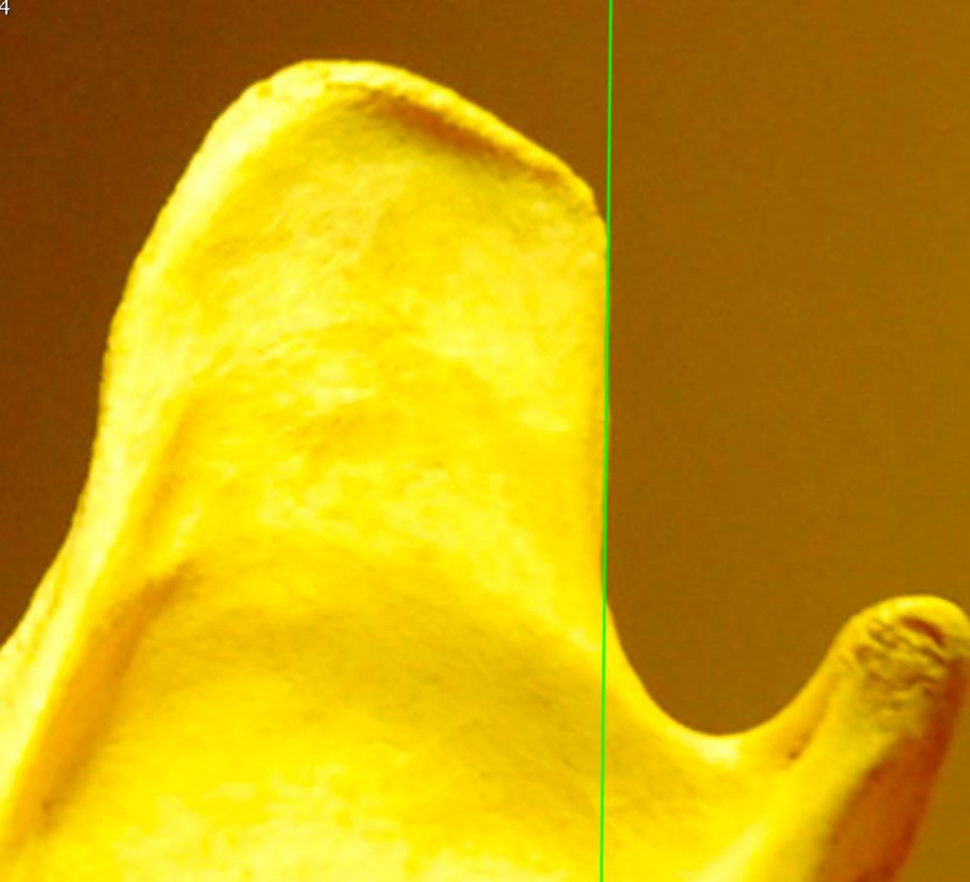
Category 1: Straight caudal border of the vertical ramus. The *vertical line* that coincides with the ventral part of the caudal border of the vertical ramus of the mandible does not cut through the dorsal caudal ramus

We tested the statement of Olsen and Olsen (1977) by examining 384 dog mandibles of many breeds, of which six breeds are Asian or American, and 60 wolf mandibles of four sub-species. Our aim is to examine whether this “turned back” morphology is indeed present in “all” dogs and only in *C. lupus chanco* as hypothesized.

## Materials and methods

All examined mandibles are from reputable museum collections. These had been collected in historical and recent periods and were professionally prepared. All are intact and from adult animals. In total 444 dog and wolf skulls were examined (888 mandibles) including 384 dog skulls and 60 wolf skulls. For the wolves (Table 1), 37 are from the collection of The George S. Wise Faculty of Life Sciences, Department of Zoology at Tel-Aviv University, Israel (ZMTAU). Thirty-two of these were *Canis lupuspallipes* and five *Canis lupusarabs*. Seven skulls were examined from the collection of the Natural History Museum in London, Great Britain (BMNH): six *C. lupusarabs,* and one *C. lupuspallipes*. Eleven skulls are from the collection of the Natural History Museum Bern, Switzerland (NMBE), all from Eurasian wolves (*Canis lupus lupus*) from Central Europe or Russia. Five specimens of *C. lupus chanco* from the collection of the Department of Vertebrate Zoology, Smithsonian Institution at the National Museum of Natural History, Washington DC, USA (USNM), were also examined.

**Table 1:** List of wolf skulls used in this study

Museum ID	Genus	Species	Sub-species	Region
BMNH ZD.1891.2.5.1	*Canis*	*lupus*	*arabs*	Bouraida
BMNH ZD.1895.10.8.1	*Canis*	*lupus*	*arabs*	Aden
BMNH ZD.1899.11.6.36	*Canis*	*lupus*	*arabs*	Muscat
BMNH ZD.1924.8.13.1	*Canis*	*lupus*	*arabs*	Jeddah
BMNH ZD.1940.193	*Canis*	*lupus*	*pallipes*	?
BMNH ZD.1948.368	*Canis*	*lupus*	*pallipes*	?
BMNH ZD.1897.1.14.4	*Canis*	*lupus*	*arabs*	Jaquakar
NMBE1028185	*Canis*	*lupus*	*lupus*	Russia
NMBE1028188	*Canis*	*lupus*	*lupus*	Russia
NMBE1028189	*Canis*	*lupus*	*lupus*	Russia
NMBE1028192	*Canis*	*lupus*	*lupus*	Poland
NMBE1028193	*Canis*	*lupus*	*lupus*	Russia
NMBE1028204	*Canis*	*lupus*	*lupus*	Poland
NMBE1028205	*Canis*	*lupus*	*lupus*	Poland
NMBE1028206	*Canis*	*lupus*	*lupus*	Poland
NMBE1028207	*Canis*	*lupus*	*lupus*	Poland
NMBE1028209	*Canis*	*lupus*	*lupus*	Poland
NMBE1028211	*Canis*	*lupus*	*lupus*	Russia
USNM00607	*Canis*	*lupus*	*chanco*	China
USNM00610	*Canis*	*lupus*	*chanco*	China
USNM00613	*Canis*	*lupus*	*chanco*	China
USNM00616	*Canis*	lupus	*chanco*	China
USNM00619	*Canis*	*lupus*	*chanco*	China
ZMTAU 09439	*Canis*	*lupus*	*pallipes*	Golan
ZMTAU 09460	*Canis*	*lupus*	*arabs*	Sandiya
ZMTAU 10334	*Canis*	*lupus*	*pallipes*	Galilei
ZMTAU 10338	*Canis*	*lupus*	*pallipes*	Galilei
ZMTAU 10355	*Canis*	*lupus*	*pallipes*	Golan
ZMTAU 10402	*Canis*	*lupus*	*pallipes*	Golan
ZMTAU 10608	*Canis*	*lupus*	*pallipes*	Galilei
ZMTAU 10609	*Canis*	*lupus*	*pallipes*	Golan
ZMTAU 10610	*Canis*	*lupus*	*pallipes*	Golan
ZMTAU 10615	*Canis*	*lupus*	*pallipes*	Golan
ZMTAU 10619	*Canis*	*lupus*	*pallipes*	Golan
ZMTAU 10621	*Canis*	*lupus*	*pallipes*	Golan
ZMTAU 10682	*Canis*	*lupus*	*pallipes*	Golan
ZMTAU 10685	*Canis*	*lupus*	*pallipes*	Golan
ZMTAU 10686	*Canis*	*lupus*	*pallipes*	Golan
ZMTAU 10688	*Canis*	*lupus*	*pallipes*	Golan
ZMTAU 10692	*Canis*	*lupus*	*pallipes*	Golan
ZMTAU 11041	*Canis*	*lupus*	*pallipes*	Galilei
ZMTAU 11109	*Canis*	*lupus*	*pallipes*	Galilei
ZMTAU 11110	*Canis*	*lupus*	*pallipes*	Golan
ZMTAU 11118	*Canis*	*lupus*	*pallipes*	Galilei
ZMTAU 11119	*Canis*	*lupus*	*pallipes*	Golan
ZMTAU 11121	*Canis*	*lupus*	*pallipes*	Golan
ZMTAU 11250	*Canis*	*lupus*	*pallipes*	Galilei
ZMTAU 11275	*Canis*	*lupus*	*pallipes*	Galilei
ZMTAU 11417	*Canis*	*lupus*	*pallipes*	Galilei
ZMTAU 11418	*Canis*	*lupus*	*pallipes*	Golan
ZMTAU 11475	*Canis*	*lupus*	*arabs*	Negev
ZMTAU 11476	*Canis*	*lupus*	*pallipes*	Golan
ZMTAU 11479	*Canis*	*lupus*	*pallipes*	Galilei
ZMTAU 11516	*Canis*	*lupus*	*pallipes*	Golan
ZMTAU 11685	*Canis*	*lupus*	*pallipes*	Golan
ZMTAU 12130	*Canis*	*lupus*	*pallipes*	Galilei
ZMTAU 12130-2	*Canis*	*lupus*	*arabs*	Negev
ZMTAU 12251	*Canis*	*lupus*	*arabs*	Negev
ZMTAU 12254	*Canis*	*lupus*	*arabs*	Muscat
ZMTAU 12279	*Canis*	*lupus*	*arabs*	Negev

Sub-species, institute and accession numbers (ID) are reported. BMNH: British Museum of Natural History. NMBE: Natural History Museum Bern, Switzerland, USNM: Department of Vertebrate Zoology, Smithsonian Institution at the National Museum of Natural History, Washington DC, USA, ZMTAU: Department of Zoology at Tel-Aviv University, Israel

We also examined 123 dog skulls from the collection of the anatomy department of the school for Veterinary Medicine, Ghent University, Belgium, and 261 skulls from the collection of The Museum of Natural History, Bern, Switzerland (total 384) (Table 2). The skulls belong to 72 different breeds, of which six breeds and 33 skulls are Asian or American. These are Alaskan malamute (5), Canadian Eskimo dog (4), Chow–Chow (16), Shar Pei (1), Tibetan Mastiff (6) and Tibetan Terrier (1).

**Table 2:** Dog skulls used in this study grouped alphabetically by breed

Breed	Nr	TB	Breed	Nr	TB
Afghan hound	13	2	Greyhound	10	1
Airedale terrier	4	1	Groenendael Belgian shepherd	18	1
**Akita** **Inu**	8	1	Hahoawu	1	
**Alaskan** **Malamute**	5	2	Irish setter	2	
Barzoi	11	2	Irish wolfhound	8	2
Basenji	1		Jagdterrier	2	
Batak hound	11	3	Karelian Bear dog	18	3
Beagle	9	2	Kuvasc	1	
Bearded collie	1		Labrador retriever	13	2
Berger de Brie	1		Leonberger	1	
Berner Sennenhund	32	4	Lundehund	2	
Bloodhound	7	1	Malinois Belgian shepherd	2	1
Border collie	5	3	Mastino Napolitano	1	
Bouvier des Flandres	4	2	Mayar Agar	2	1
Boxer	2		Pariah hound	10	2
Bull terrier	1		Pembroke Welsh Corgi	1	
Canaan dog	1		Pharaoh hound	4	
**Canadian Eskimo** **dog**	4		Pointer	1	1
**Chow** **Chow**	16	3	Poodle	6	2
Cocker spaniel	4		Rhodesian Ridgeback	2	2
Crossbred	5	3	Rottweiler	3	
Dalmatian	1		Saint Bernhard	2	
Dingo	3	2	Saluki	2	
Doberman pinscher	15	5	Samojeed	8	2
Entelbucher	1		Scottish collie	1	
Finnish spitz	3	1	Scottish terrier	16	
Flatcoat retriever	1		**Shar** **Pei**	1	
Fox terrier	1		Siberian Husky	14	3
Gaint schnauzer	1		Sloughi	1	
Galgo Espanjol	2		Swiss shepherd	1	
German braque	3	1	Tervueren Belgian shepherd	5	
German shepherd	10	3	**Tibetan** **Mastiff**	6	1
Golden retriever	6	1	**Tibetan** **spaniel**	1	
Great Dane	2		Weimaraner	1	
Great spitz	7	2	Whippet	4	2
Greenland dog	10	1	Wolfspitz	2	1
Total breeds	72		Total skulls	384	

In bold are New World and Asian breeds. Nr refers to the number of skulls examined. TB refers to “Turned Back” morphology

**Fig. 2:**
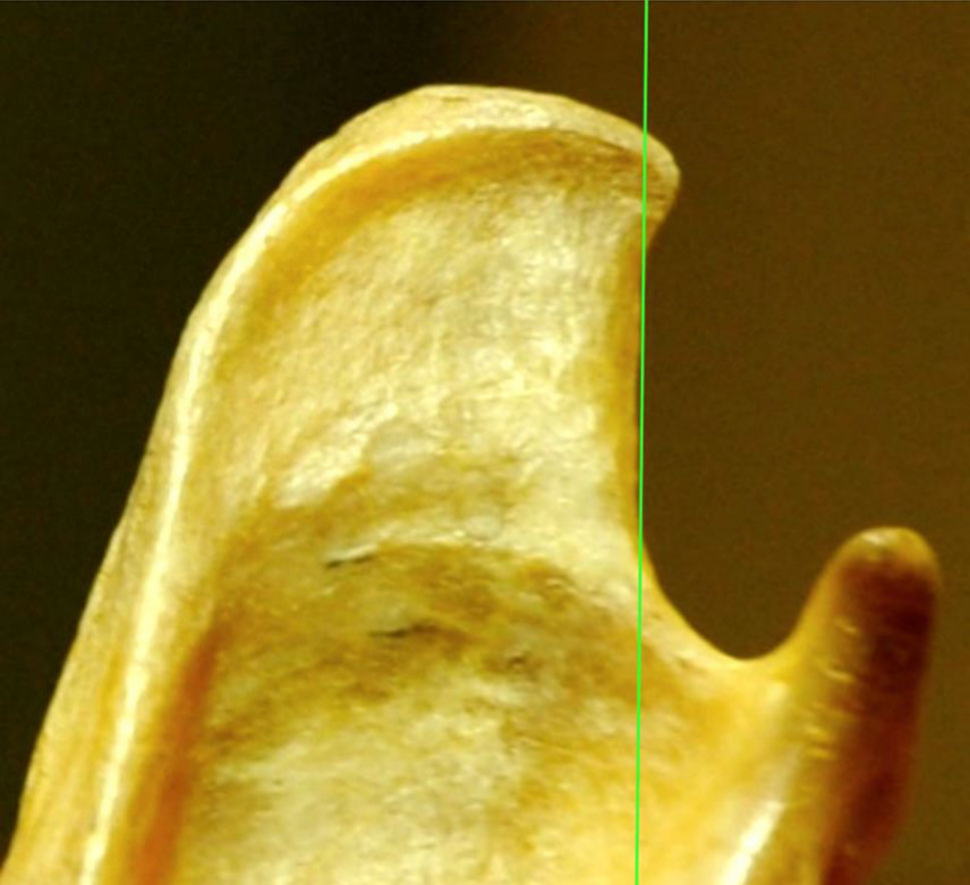
Category 2: Straight caudal border with minimal tip curvature. The *vertical line* that coincides with the ventral part of the caudal border of the vertical ramus of the mandible coincides with the caudal border and does only cut through the tip of dorsal caudal ramus

**Fig. 3:**
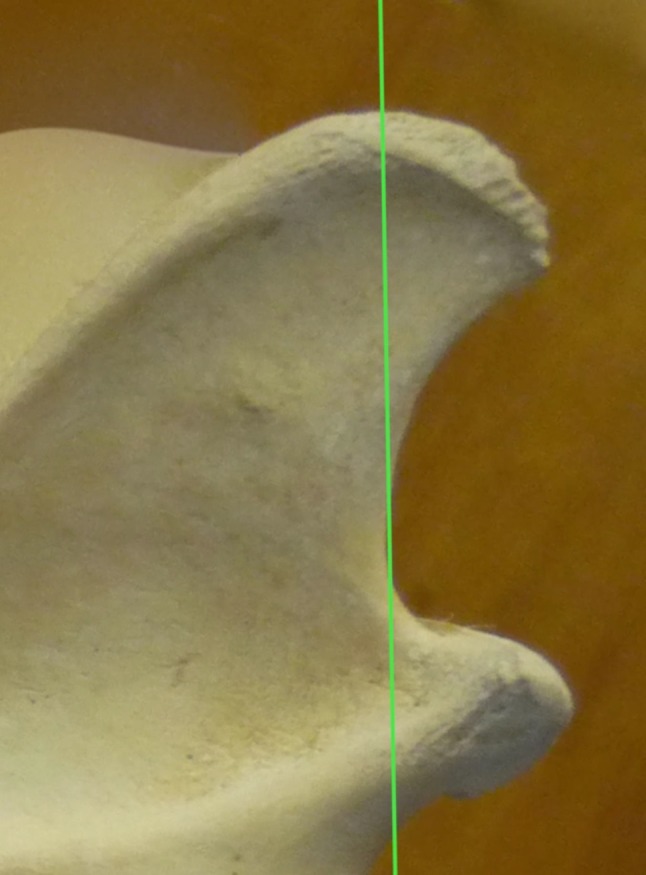
Turned back morphology. The *vertical line* at the caudal border of the vertical ramus of the mandible does not coincide with the border and cuts through a large part of the dorsal ramus

Each mandible was digitally photographed from a distance of 40–50 cm with a digital Nikon D 700 camera with a 50 mm lens. The photographs were imported in the OsiriX Imaging Software program. A straight vertical line was then drawn confluent with the straight part of the ventral caudal border of the mandible. The mandibles were divided in two categories based on the morphology of the coronoid process and by drawing a straight line (green on the figures) coinciding with the caudal border. For Category 1, the mandible has a perfect vertical straight caudal border (Fig. 1) or the uppermost part of the apex points minimally in the caudal direction, while the caudal border is straight (Fig. 2). In this category, the straight green line follows the caudal bony border of the vertical ramus and the dorsal aspect of the mandible does not cross the green line or transects only a very small part at the tip. For Category 2, the caudal border is concave over its entire length and has the form of a dolphin fin (Fig. 3). Here, the vertical line transects most of the caudal vertical ramus and the line cannot coincide with the caudal border which is concave.

## Results

**Fig. 4:**
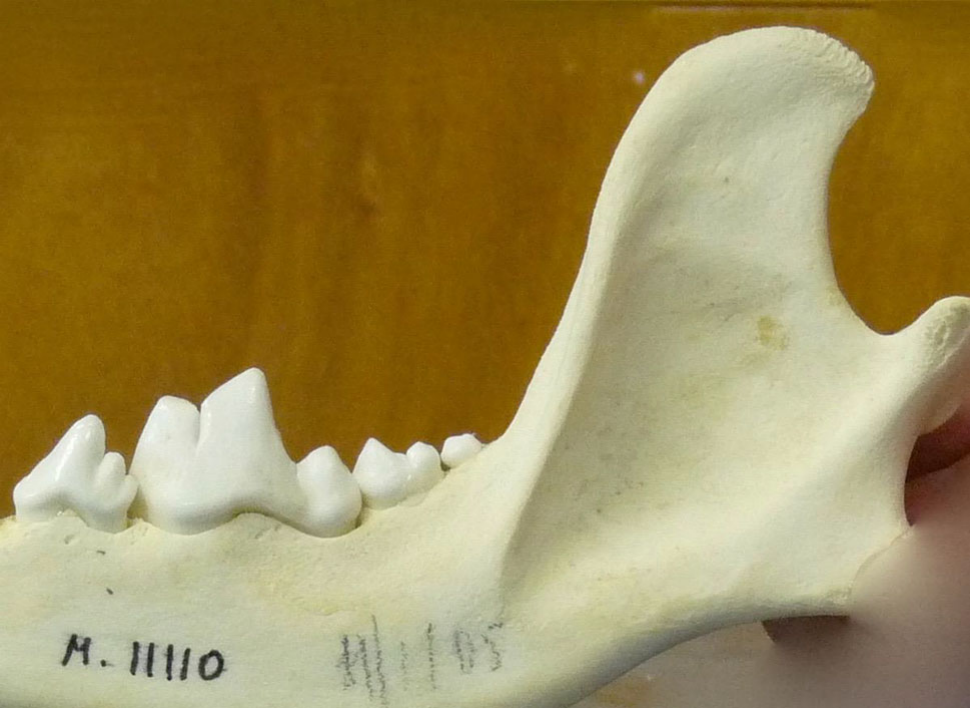
A *Canis lupus pallipes* mandibular specimen with “turned back” morphology. Accession number ZMTAU1110 (George Wise faculty of Life Sciences, Israel)

**Fig. 5:**
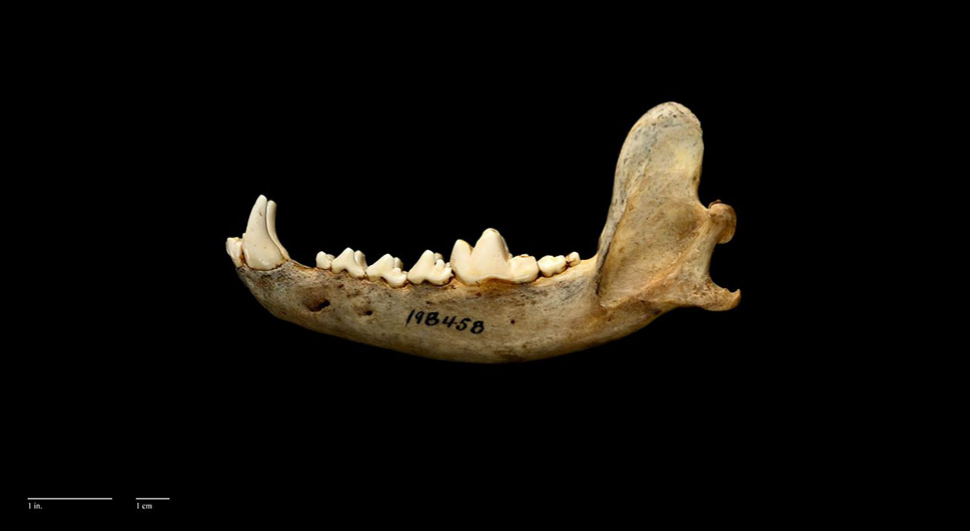
The *Canis lupus chanco* mandibular specimen without the “turned back” morphology. Accession number 18B458- NHB 2015- USNM00610 (Smithsonian Institution, USA). Photo: D. E. Hurlbert

All left and right mandibles from the same skull show identical anatomy; therefore, frequencies are per skull, not mandible. Fifty-two wolf skulls had a straight caudal border (87 %), while eight (13 %) had a “turned back” morphology (Table 3). Eurasian wolves and *C. lupus arabs* all had straight mandibles. *C. lupus pallipes* had four specimens with mandibles with the “turned back” morphology (12 %) (Fig. 4) and C. *lupus chanco* four out of five mandibles with “turned back” morphology (80 %) but one with straight morphology (20 %) (Fig. 5).

**Table 3:** Morphological categories of the coronoid process of the mandible

	Dogs	*Canis lupus*	*Canis lupus*	*Canis lupus*	*Canis lupus*	Wolves
		*pallipes*	*arabs*	*chanco*	Eurasian	Total
Total number	384	37	7	5	11	60
Category 1: straight morphology	81 % (312)	88 % (33)	100 % (7)	20 % (1)	100 % (11)	52
Category 2: “Turned back” morphology	19 % (72)	12 % (4)		80 % (4)		8

Of the 384 dog skulls, 312 had a straight caudal border (81 %) and 72 mandibles had “turned back” morphology (19 %). There was no relation between the “turned back” anatomy and breed; this was spread across 37 breeds (Table 3).

**Fig. 6:**
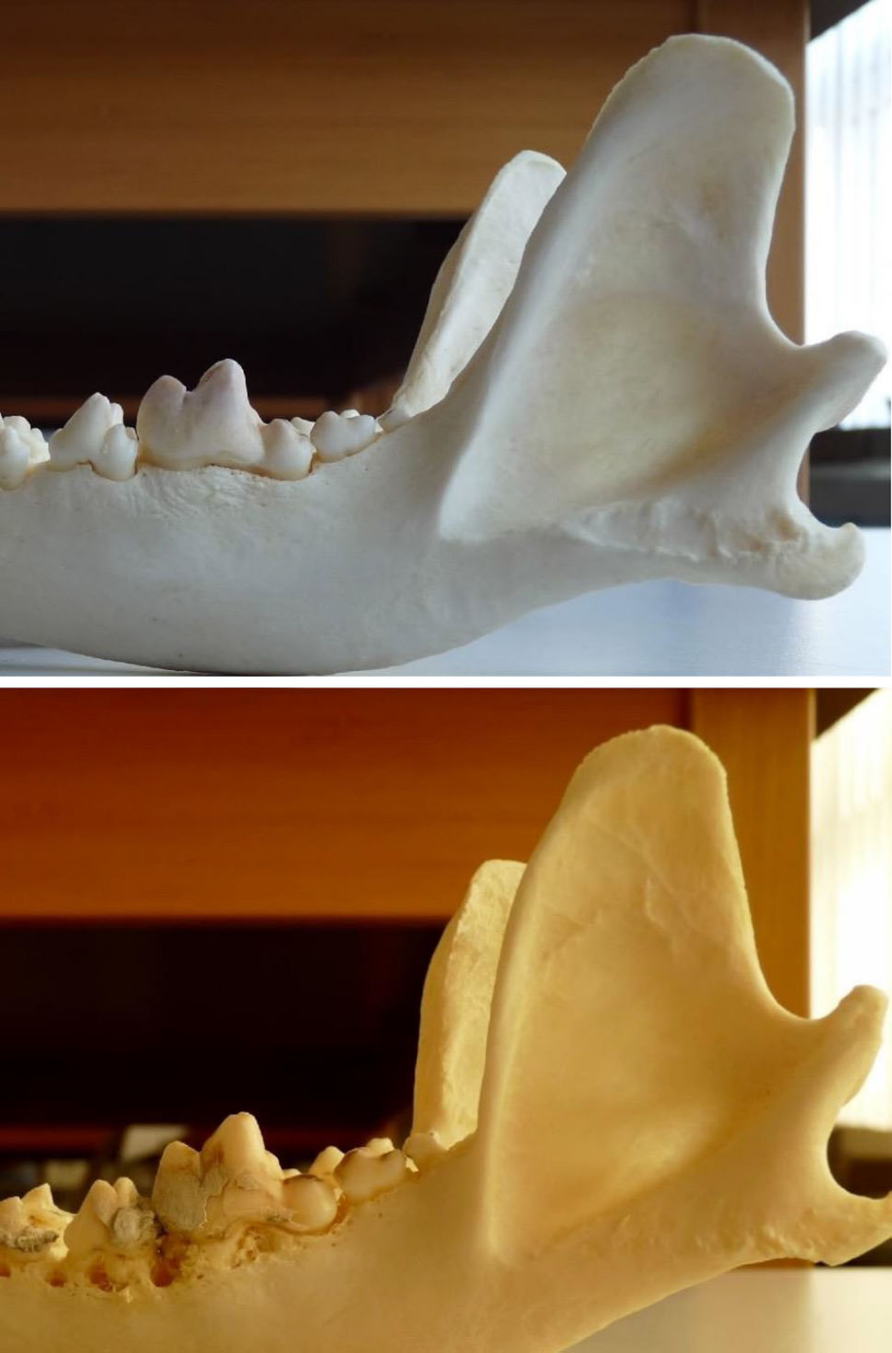
A mandibular specimen of an Asian/American dog without the “turned back” morphology. *Top* Alaskan Malamute specimen. Accession number 1051378-313/78 (Museum of Natural History, Bern, Switzerland). *Bottom* Akita Inu specimen. Accession number 1051382-523/82 (Museum of Natural History, Bern, Switzerland)

Three of seven Asian and American breeds (41 mandibles) had seven “tuned back” mandibles (17 %) so most mandibles in these breeds were straight (Fig. 6).

## Discussion

Three main claims are made in Olsen and Olsen’s article (1977). The first is that the Chinese wolf is progenitor to Asian and New World dogs. When Olsen and Olsen’s article (1977) was published, it was still uncertain if only the wolf was a progenitor to dogs. In addition to the wolf, *Canis aureus* was said to be a possible forefather of small breed dogs (Darwin 1868; Lorenz 2002). It was also uncertain if there had been only one domestication wave, or if regional and different domestication phenomena had occurred and so for example local Asian wolves could then have been directly ancestral to Asian and New World dogs and Eurasian wolves to European dogs. The article should thus be viewed in this historical perspective. The fact that *C. lupus chanco* is called “the Chinese wolf” in the article, not Tibetan wolf (Pocock 1946), should also be placed in the same historical perspective as the 1970s were a period of a Sino-American rapprochement (Oksenberg 1982). Recent genetic analysis has confirmed that only wolves are progenitors to dogs, contradicting older theories about different geographic domestication waves (Duleba et al. 2015; Horard-Herbin et al. 2014; Larson et al. 2012; Thalmann et al. 2013) and has revealed that New World dogs did not originate locally but invaded the continent together with early migration waves of *Homo sapiens* (Leonard et al. 2002; Savolainen et al. 2002).

The original article shows drawings of 13 mandibles of which only ten have sufficient intact anatomy to make interpretation possible (according to personal re-examination of the published drawings by LJ). Of these, six belong to dogs, one to *C. lupus chanco* and three to species other than *Canis lupus*. All dogs and all *C. lupus chanco* specimens show the “turned back” anatomy. It is not reported if more than these seven mandibles were examined. If not, it is difficult to understand why such a general statement was published. *C. lupus chanco* skulls are very difficult to find in zoological and natural history collections. This may explain why only one was reported in the article. We found only eleven skulls in many worldwide collections. Of these only five had intact mandibular anatomy, of which one (20 %) had a straight caudal mandibular ramus, contradicting Olsen and Olsen’s (1977) original statement.

The second assertion is that the “tuned back” morphology is absent from other canids. This statement is unsupportable as we have demonstrated the presence of the “turned back” morphology in *C. lupus pallipes* mandibles. Studer (1901) early on reported that from all examined wolf skulls *pallipes* and *chanco* were the most anatomically similar. This may explain why these two wolf sub-species share this “turned back” morphology, unseen in the two other wolf sub-species we examined.

The third statement is that “dogs have the turned back morphology”. At one point in the article this statement is made in general: “all dogs” have the turned back morphology (Olsen and Olsen 1977, 534, last paragraph), while in another location it refers to “New World and Asian dogs” (Olsen and Olsen 1977, 533, fifth paragraph), while the title of the article refers only to New World dogs. The “turned back” morphology is present in the six dog mandible drawings in the article, but the same pattern was not observed in the large group of dog mandibles we examined, not in general and not in Asian or New World dogs. Indeed, only a minority of dogs (20 %) have “turned back” morphology. In addition there are no differences in occurrence between Asian and/or New World dogs nor in the total group of dogs (18 % in these breeds vs. 20 % in total).

## Conclusion

The statement that all dogs have a specific “turned back” morphology of the mandibular coronoid process, and that they share this specific morphology with only one wolf sub-species (*C. lupus chanco*), is untenable. This morphological trait cannot therefore be used as an argument to claim that archaeological remains belong to dogs, nor to argue that *C. lupus chanco* is the progenitor of dogs.
